# Adult Tissue Extracellular Matrix Determines Tissue Specification of Human iPSC‐Derived Embryonic Stage Mesodermal Precursor Cells

**DOI:** 10.1002/advs.201901198

**Published:** 2020-01-21

**Authors:** Imran Ullah, Jonas Felix Busch, Anja Rabien, Bettina Ergün, Christof Stamm, Christoph Knosalla, Stefan Hippenstiel, Petra Reinke, Andreas Kurtz

**Affiliations:** ^1^ Berlin Institute of Health Center for Regenerative Therapies Charité Universitätsmedizin Berlin Augustenburger Platz 1 13353 Berlin Germany; ^2^ Department of Urology Charité–Universitätsmedizin Berlin 10117 Berlin Germany; ^3^ Berlin Institute for Urologic Research 10117 Berlin Germany; ^4^ Deutsches Herzzentrum Berlin and German Center for Cardiovascular Research Augustenburger Platz 1 13353 Berlin Germany; ^5^ Department of Infectiology and Pneumonology Charité–Universitätsmedizin Berlin Augustenburger Platz 1 13353 Berlin Germany

**Keywords:** cell differentiation, extracellular matrix, pluripotent stem cells, regenerative medicine, tissue engineering

## Abstract

The selection of pluripotent stem cell (PSC)‐derived cells for tissue modeling and cell therapy will be influenced by their response to the tissue environment, including the extracellular matrix (ECM). Whether and how instructive memory is imprinted in adult ECM and able to impact on the tissue specific determination of human PSC‐derived developmentally fetal mesodermal precursor (P‐meso) cells is investigated. Decellularized ECM (dECM) is generated from human heart, kidney, and lung tissues and recellularized with P‐meso cells in a medium not containing any differentiation inducing components. While P‐meso cells on kidney dECM differentiate exclusively into nephronal cells, only beating clusters containing mature and immature cardiac cells form on heart dECM. No tissue‐specific differentiation of P‐meso cells is observed on endoderm‐derived lung dECM. P‐meso‐derived endothelial cells, however, are found on all dECM preparations independent of tissue origin. Clearance of heparan‐sulfate proteoglycans (HSPG) from dECM abolishes induction of tissue‐specific differentiation. It is concluded that HSPG‐bound factors on adult tissue‐derived ECM are essential and sufficient to induce tissue‐specific specification of uncommitted fetal stage precursor cells.

## Graphical abstract

The extracellular matrix holds tissue‐specific memory, which can trigger efficient tissue‐specific differentiation of lineage committed cells. Cues responsible for the specification are conserved throughout development and depend on the presence of heparan sulfate proteoglycans (HSPGs). Modulation of HSPG‐decorating factors can be utilized for matrix engineering.

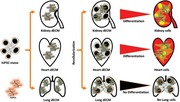

## 1 Introduction

The control of interactions between cells and their environment is essential for tissue and disease modeling, and regenerative medicine. The extracellular matrix (ECM) serves as a key messenger between cells and the tissue environment by providing a dynamic scaffold. ECM‐embedded cues regulate organ homeostasis, meditate cellular responses, and promote tissue repair.^1^ These cues are not only organ‐specific but also dynamically changing during tissue morphogenesis and maturation. Isolated ECM may not only provide a structural scaffold of the source organ, but also a developmental stage and tissue‐specific functional imprint that could be used as a basis for generating organ models.^2^ For example, decellularized ECM (dECM) from heart, kidney, lung, and other organs has been used as scaffold for recellularization with tissue‐specific cells, non‐committed stem and progenitor cells to reestablish cell functionality and to study cell–ECM interaction with the vision to ultimately engineer organs for transplantation.^3^, ^4^, ^5^, ^6^ The use of pluripotent stem cells (PSC) as a cell source for dECM recellularization was based on the assumption, that the ECM memorizes its tissue origin and provides cues, which drive organotypic differentiation processes.^6^, ^7^, ^8^, ^9^, ^10^ Indeed, expression of organ‐specific cell markers was detected in renal, pancreatic, or cardiac dECM recellularized with undifferentiated PSC, however, reproducibility of obtaining structurally or functionally mature cells was usually poor.^6^, ^7^, ^8^, ^9^, ^10^ More efficient and reproducible differentiation was shown with a tissue specifically committed progenitor cell population for recellularization of dECM from different organs.^8^, ^11^ These data indicate that the developmental stage at which cells respond to dECM may be of importance. This is relevant as most PSC‐derived cells are immature.^12^ Furthermore, for cell therapy, the most efficient tissue repair and regeneration may be achieved with proliferative active and plastic progenitor cells instead of terminally differentiated cells. However, these cells have to be able to respond to the adult, aged, or compromised environment, including the ECM. As reviewed, previous results from human and mouse lung^2^, ^14^, ^15^ indicate that the adult ECM contains memory factors and cues that are able to promote tissue‐specific differentiation of tissue‐committed stem cells, very little is known about the nature and identity of the functional cues driving this process.^13^ However, whether these ECM‐associated cues are conserved between species is unclear and using cells and ECM from different species may confound results. Strong evidence was provided that heparan sulfate proteoglycans (HSPG) are required to drive epithelial differentiation of endoderm cells on lung dECM as their removal diminishes differentiation.^14^, ^15^

Moreover, in most studies to elucidate dECM effects on stem cells, differentiation promoting factors were provided with the cell cultivation medium. The presence of these factors makes it difficult to assess the specific role of ECM and ECM‐bound molecules for the observed cell specification effects. In fact, externally supplemented inducing factors also work efficiently with undefined matrices such as mouse tumor‐derived matrigel to promote differentiation of human stem cells.^16^ Whether target tissue‐derived ECM may have any additional differentiation, promoting and specifying effects is uncertain and was not directly compared to matrigel. Moreover, the property of autonomous self‐organizing morphogenesis of more committed precursors, for example, of metanephric mesenchyme precursor cells of the kidney,^8^ may additionally blur the role and need of exogenous ECM as a promoter of tissue‐specific differentiation.^17^, ^18^ Finally, the use of both dECM and cells from human will eliminate variability introduced by cross species approaches when investigating specification of early human precursor cells in a human environment.

Here, we aimed at elucidating whether organ‐specific imprinting of mature adult ECM provides essential and sufficient cues for tissue‐specific differentiation of uncommitted embryonic stage human mesodermal precursor cells. To minimize experimental variables such as species differences, medium components, decellularization methodologies, cell source, and differentiation stage, we established a humanized system, where these variables are controlled and standardized. In addition, we aimed at elucidating the nature of the ECM cues responsible for inducing organotypic cell specification and whether structural factors may also trigger differentiation. The results show that dECM retains distinct tissue‐specific memory, which is imprinted in adult ECM‐decorating heparin‐binding growth factors and act on immature embryonic stage precursor cells.

## 2 Results and Discussion

### 2.1 Differentiation Specificity of Human Induced PSC‐Derived Mesodermal Precursor Cells (P‐meso) Depends on Tissue Origin of Decellularized ECM

The use of ECM as a bioactive matrix for cells in vitro and in vivo is based on its interactive properties and tissue‐specific imprints, which are recognized as cues by cells to adopt tissue‐specific phenotypes. For example, decellularized kidney, lung, heart, or skeletal muscle ECM, have been used for recellularization with pluripotent stem cells, tissue committed precursor and mature cell types. Assessment of the resulting phenotypic patterns revealed in most cases variable proportions of tissue‐specific cell types.^3^, ^4^, ^13^, ^17^, ^19^, ^20^, ^21^, ^22^, ^23^ However, the addition of differentiation‐inducing factors in the culture media, and the use of non‐human and non‐standardized dECM preparations made it difficult to distinct the role of the ECM in the observed organotypic determination.^24^, ^25^ Here, we eliminated confounding variables to determine the potency of intrinsic imprinting of ECM. We omitted inductive factors within cultivation media, applied a standardized decellularization method for all tissues used, and used only human ECM and cells. Moreover, we tested early fetal stage not yet tissue‐committed mesoderm precursor cells on ECM from mature adult tissues most likely encountered by cells in regenerative medicine and in vitro modeling. Finally, we directly compared the effects of human kidney, heart, and lung ECM on these cells.

**Figure 1:**
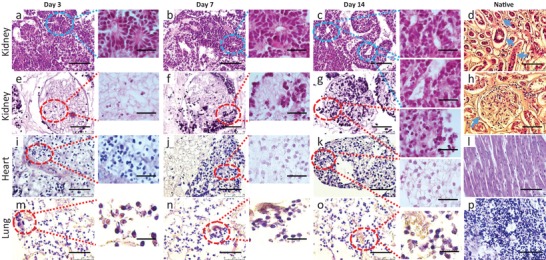
Histological characterization of kidney, heart and lung dECM repopulated with P‐meso cells. Representative hematoxylin and eosin (H&E) staining of kidney, heart, and lung scaffold sections on days 3, 7, and 14 post seeding with P‐meso cells, and of respective native tissue sections. Overview of: a–c,e–g) recellularized kidney, i–k) heart, and m–o) lung dECM. b) Circles indicate circular and longitudinal tubule/vessels‐like structures. c) Tubule/vessel‐like structures (circles) appear more compact compared to day 14. d) Native human kidney. Circles indicate blood vessel and tubular structures. e–g) Higher magnification of a renal glomerulus in dECM (circles). g) Cells arrange similar to native glomerulus seen in (h). j) P‐meso cells on heart dECM clustered at day 7, when beating cells were first observed. k) Clusters increased in size and number by day 14 with continues beating. l) Native human heart. m–o) Cells scattered on lung dECM with no structural changes over time. p) Native human lung. Seven independent experiments were performed for each tissue with three different hiPSC‐lines. Scale bar: 75 and 20 µm.

**Figure 2:**
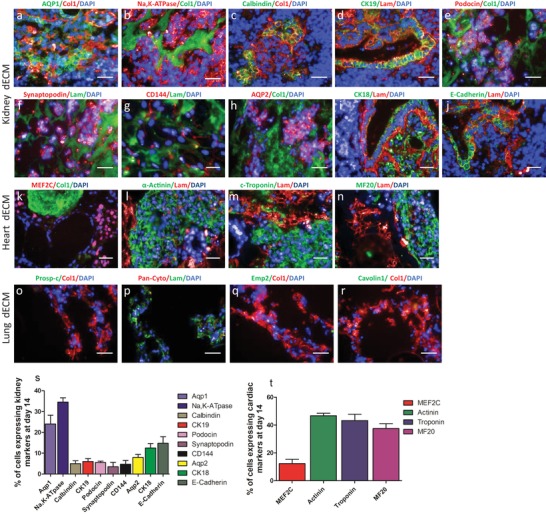
Immunohistochemical characterization of hiPSCs‐derived P‐meso cells on kidney, heart, and lung dECM. a–j) Kidney, heart, and lung cell markers were selected to identify different renal cell types, k–n) immature and mature cardiac cells, and o–r) different lung epithelial cell types: a,b) AQP1 and Na,K‐ATPase for proximal tubules, c) Calbindin for distal tubules, d) CK19 for loop of Henle, e,f) podocin and synaptopodin for glomerulus, g) CD144 for endothelial cells, h) AQP2 for collecting duct, i,j) CK18 and E‐Cadherin for nephron epithelia. k) MEF2C as cardiac progenitor marker, l–n) α‐actinin, c‐Troponin and MF20 as more mature cardiomyocyte markers, o,p) lung epithelia, q) alveolar type‐II cells, r) multiple lung cell types. Images are from different depths of the 800 µm thick ECM showing uniform cellular penetration. Scale bar: 20 µm. *n* = 7. s,t) Percentage of cells expressing kidney (s) and heart (t) markers at day 14 post differentiation induction. ± SEM, *n* > 2.

Human kidney, heart, and lung tissues were decellularized and analyzed for preservation of structure and tissue architecture. For characterization, the 800 µm thick dECM slices were attached to glass slides for stabilization, improved handling, and reproducible analysis. Maintained collagen and laminin structures and absence of nuclear stain indicated complete cell removal and maintenance of major structural properties (Figure S1, Supporting Information). To investigate the effects of the tissue origin of the dECM on cell differentiation, human induced pluripotent stem cells (hiPSC) were differentiated into P‐meso, characterized by the uniform expression of T (Brachyury), HAND1, and goosecoid^26^ and absence of endodermal and pluripotency marker expression (Figure S2, Supporting Information). The P‐meso cells were seeded on dECM from healthy adult kidney, heart, and lung tissue in an air‐liquid interphase (ALI) cultivation system in growth factor‐free medium for 14 days. We expected that mesoderm precursor cells may recognize marks on adult ECM‐derived from tissues of mesoderm origin such as kidney and heart, but not on dECM from endoderm‐derived tissues such as the lung. On kidney dECM, the cells were initially scattered, uniformly distributed, and some organization patterns were observed by day 3 (**Figure** **1**a,e). By day 7, cells organized and formed tubular structures reminiscent of those found in nephron‐forming elements during renal development (Figure 1b,f). Patterning continued until day 14, when the cells became more arranged, tubular elements were more prominent, rounded structures were increasing in number, and more elongated tubular structures appeared. Cells organized at the border of tubule‐ and blood vessel‐like matrix structures and densely grouped within glomerular areas (Figure 1c,g). The renal cells started to express typical markers of differentiated nephronal cells, including epithelial cells of the proximal and distal tubules, loop of Henle, collecting duct, glomerular podocytes, and endothelial cells by day 7 (Figure S3a–j, Supporting Information). Expression of these markers was maintained until day 14 with increasing cell numbers and structural patterning (**Figure** **2**a–j).

**Figure 3:**
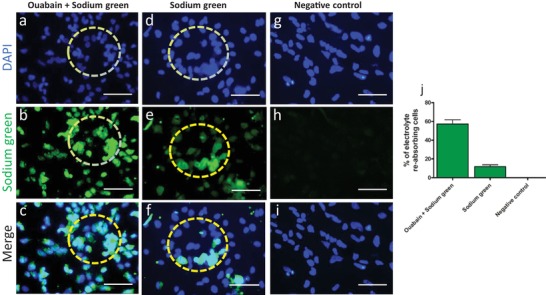
Electrolyte reabsorption hiPSC‐meso‐derived cells on kidney dECM (day 14). a–c) Sodium‐green fluorescence demonstrates sodium uptake as observed by the intracellular fluorescence signal within tubular‐like structures (circles). d–f) Ouabain inhibition of Na, K‐ATPase increased intracellular sodium levels. g–i) No fluorescence was detected when sodium‐green was omitted. j) Percentage of cells absorbing electrolytes. Scale bar: 75 µm mean ± SEM, *n* = 2.

To determine whether the P‐meso‐derived renal proximal tubular cells on kidney dECM have the capability of electrolyte reabsorption, we performed sodium uptake analysis. Exposure of the cells to ouabain enhanced sodium uptake by inhibiting Na, K‐ATPase in most of the cells (**Figure** **3**).

Spreading and organization of the P‐meso cells on heart dECM was distinctly different from the pattern observed on kidney dECM (Figure 1i–k). On day 3, in heart, dECM the cells were evenly scattered and accumulated into cell condensates by day 7, which started to beat (Video S1, Supporting Information) and to express typical markers of cardiomyocytes from day 7 (Figure S3k–n, Supporting Information) until at least day 14 (Figure 2k–n), including the cardiac progenitor marker Myocyte enhancer factor 2C (MEF2C) and markers of more mature cardiac cells c‐troponin, α‐actinin, and myosin. Cell condensates were maintained by day 14 with increasing numbers of beating cell clusters (Figure 1k), which were stable at least until day 30 when the experiment was terminated (not shown).

**Figure 4:**
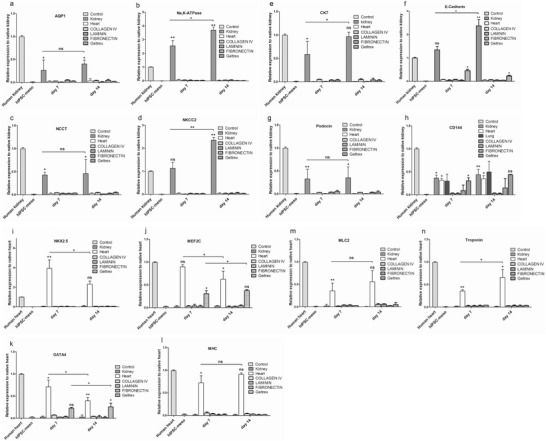
Transcription of renal and cardiac markers in P‐meso‐derived cells on kidney, heart dECM, and single matrix proteins collagen IV, laminin, fibronectin, and geltrex. RNA expression analysis by qPCR reveals increased expression from day 7 to day 14 of tissue‐specific renal transcripts AQP1, Na,K‐ATPase, NCCT, CK19, AQP2, E‐Cadherin, and podocin only in cells on kidney dECM (a–h), and of cardiac transcripts NKX2.5, MEF2C, GATA 4, MHC, MLC2, and troponin only in cells on cardiac dECM (i–n). h) The endothelial marker CD144 was expressed in cells on kidney, heart, and lung dECM. f) On geltrex, only E‐Cadherin was induced in P‐meso cells and detected at days 7 and 14. i) Similarly, the endothelial marker CD144 was induced by geltrex. j–n) The cardiac markers MEF2C and GATA4 were induced on geltrex on days 7 and 14 post seeding. Gene expression was normalized to the native human tissue, mean ± SEM, *p < 0.005, *n* = 7.

In contrast, the P‐meso cells on lung dECM spread uniformly over the matrix and proliferated but did not show any differentiation pattern (Figure 1m–o) or expression of the lung epithelial cell markers Prosurfactant Protein C (proSp‐C), Pan‐cytokeratin, Epithelial membrane protein 2 (EMP2), and Caveolin1 until day 14 (Figure 2o–r; Figure S3o–r, Supporting Information). This corroborates our assumption that ECM of endoderm‐derived lung tissue is unable to support and promote mesoderm‐lineage specification and is unable to transdifferentiate iPSC‐derived mesoderm precursors into lung epithelial cells. However, CD144 positive endothelial cells, which are of mesoderm origin, were induced from the P‐meso cells on all three matrices (**Figure** **4**h). The percentage expressions of different renal and cardiac markers at day 14 were quantified (Figure 2s–t).

To elucidate whether cells integrate into the full depth of the 800 µm thick dECM slices, analysis of an average of ninety 5 µm sections taken from various tissue depths from recellularized kidney, heart, and lung slices demonstrated cell penetration throughout the full matrix thickness (Figures 1 and 2; Figures S3 and S4, Supporting Information). To confirm selective differentiation of P‐meso cells on cardiac and renal dECM, mRNA expression of renal, cardiac, and endothelial markers was determined by quantitative real‐time polymerase chain reaction (qPCR) on days 7 and 14 post seeding (Figure 4a–n). Interestingly, expression of immature cardiac marker genes homeobox gene NKX2.5, Myocyte enhancer factor 2 (MEF2C), and the zinc‐finger transcription factor GATA4 were expressed higher on day 7, with declining expression by day 14 (Figure 4i–k), while expression levels of the more mature cardiac markers myosin light and heavy chain and cardiac troponin increased between days 7 and 14 (Figure 4l–n). Only CD144 positive endothelial cells, which are of mesoderm origin, were induced from the P‐meso cells on dECM of all three tissues (Figure 4h).

To assess whether the matrix proteins Col IV, Laminin, Fibronectin, or the murine tumor‐derived matrigel (geltrex) are able to promote differentiation of P‐meso cells, we seeded the cells on these individual proteins and on geltrex. As for the dECM, the cells were cultured under ALI conditions without supplementing the media with differentiation‐inducing growth factors. Expression of renal, cardiac, and lung‐specific marker genes at days 7 and 14 was determined by qPCR. Low expression of the selected genes was detectable on any of the single matrix proteins. On geltrex, the cardiac markers GATA4 and MEF2C, the epithelial marker E‐Cadherin and the endothelial marker CD144 were expressed at days 7 and 14 (Figure 4).

Interestingly, the distinct ability of kidney and heart dECM to differentiate early mesoderm into nephronal and cardiac cells was completely lacking on isolated matrix proteins such as Fibronectin, Laminin, and ColIV, heavily diminished on the murine tumor‐derived geltrex and absent on lung dECM. On lung dECM, the mesoderm cells failed to differentiate into lung cells, which derive from the endoderm lineage. However, mesoderm‐derived endothelial cells were readily detected in lung dECM. Interestingly, the use of porcine kidney dECM also induced differentiation of human P‐meso cells into cells of the renal lineage, however, expression of markers was reduced and not all renal cell types were detected (data not shown). These data indicate that the specific dECM decoration rather than common structural matrix proteins are responsible to germ‐line sensitive cell differentiation.

To further confirm tissue specificity of differentiation, we tested the expression of kidney markers on heart dECM and of cardiac markers on kidney dECM on days 7 and 14 post seeding with P‐meso cells. No expression of cardiac cell markers on kidney dECM or of renal cell markers on heart dECM was observed. Similarly, no renal or cardiac cell markers were expressed on lung scaffolds (Figure S4, Supporting Information).

Differentiation and specification of P‐meso cells into nephronal and cardiac cells using human kidney and heart dECM were reproduced with three different human iPSC‐lines: WISCi004‐A (IMR90), BCRTi005‐A, and BIHi004‐A (Figure S2, Supporting Information).

The mesodermal markers‐expressing hiPSC‐derived P‐meso cells arise early in human embryogenesis and are plastic mesendodermal precursor cells committed to develop into a wide range of mesodermal cell types, including muscle, kidney, endothelia, and connective tissues. We posited that these cells are better suited than pluripotent cells or further tissue committed renal, cardiac, or endothelial precursor cells to elucidate the influence of adult ECM on cellular plasticity. Indeed, kidney dECM alone directed differentiation of these P‐meso cells into a variety of nephronal cells of the main structural components, including glomerulus, proximal and distal tubules, loop of Henle, collecting duct, and endothelial cells. Other studies using pluripotent hiPSC or embryonic stem cells (hESC) or kidney‐committed metanephric mesenchyme cells also detected the expression of some renal markers when seeded on kidney dECM. While hPSC differentiate also into renal cells, the efficacy and yield is variable and not quantitatively and qualitatively reproducible. The rather tissue restricted metanephric mesenchyme cells, in connection with inductive media components differentiated preferentially into renal tubular cells.^20^, ^27^, ^28^, ^29^ A direct comparison on dECM from other tissues was not performed. We showed that heart dECM directed the P‐meso cells to cardiac progenitor and mature cells as early day 7 of culture in the absence of specific inducers with increasing expression of mature markers and reduction of early maturation markers over time. This was achieved with notable morphological and functional patterning in the used 3D in vitro dECM platform, where the P‐meso cells penetrated the full layer of the 800 µm thick dECM sections. Our defined and standardized 3D‐ECM model is thus generally suitable to investigate cell‐matrix signaling and offers an approach to efficiently generate renal and cardiac cells from hiPSC.

### 2.2 Removal of HSPG from dECM Abolishes dECM Induced Tissue‐Specific Differentiation of P‐meso Cells

**Figure 5:**
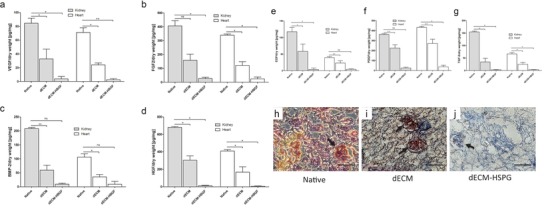
HBGF concentrations in native kidney and heart tissues, dECM, and dECM‐HSPG. a–g) Detection of indicated HBGFs in native kidney, kidney dECM, and dECM‐HSPG by ELISA. h–j) VEGF detection in kidney dECM, native kidney, and dECM‐HSPG sections by immunohistochemistry. Arrows indicate glomeruli, with strongest VEGF expression. Masson trichrome staining shows collagen (blue). Mean ± SEM, *p < 0.005, *n* = 3.

HSPGs are ECM associated glycoproteins with the common characteristic of containing one or more covalently attached heparan sulfate (HS) chains.^30^ HSPGs non‐covalently bind a number of chemokines, cytokines, enzymes, growth factors, or other bioactive molecules.^30^ We hypothesize that HSPG‐binding heparin‐binding growth factors (HBGF) may be responsible for the observed tissue‐specific memory of dECM. When we assessed the presence of HBGFs vascular endothelial growth factor (VEGF), fibroblast growth factor 2 (FGF‐2), bone morphogenetic protein 2 (BMP‐2), hepatocyte growth factor (HGF), epidermal growth factor (EGF), platelet‐derived growth factor‐beta (PDGF‐BB), and transforming growth factor‐beta (TGF‐ß), these were all detectable in ECM even after decellularization, albeit at reduced levels compared to native tissues (**Figure** **5**a–g). Heparitinase treatment to eliminate HSPGs removed these dECM‐bound HBGFs as shown exemplary for VEGF. Assessment of VEGF retention by immunostaining confirmed typical distribution patterns with highest VEGF concentrations in glomerular renal structures, which was partially maintained after decellularization and eliminated after heparitinase treatment, while the structural dECM proteins were preserved (Figure 5h–j).

**Figure 6:**
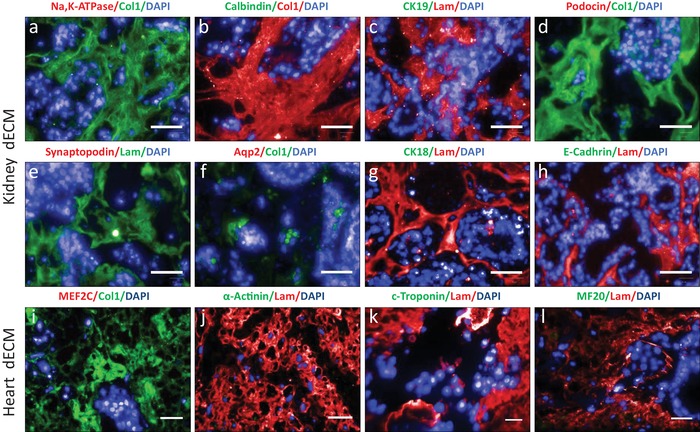
Removal of HSPGs abolishes induction of tissue‐specific cell types from P‐meso cells on kidney and heart dECM‐HSPG. Kidney and heart cell markers were used to identify different renal structures and immature and mature cardiac cell types at day 14 post seeding of P‐meso cells on dECM‐HSPG. a–h) No expression of kidney markers was detected for Na,K‐ATPase (proximal tubules), Calbindin (distal tubules), CK19 (loop of Henle), podocin and synaptopodin (glomerular podocytes), AQP2 (collecting duct), CK18, and E‐Cadherin (nephron epithelia). i–l) No expression was detected of cardiac markers MEF2C (cardiac progenitors), α‐Actinin, c‐Troponin, and MF20 (mature cardiomyocytes). Scale bar: 20 µm.

Recellularization of heparitinase‐treated dECM (dECM‐HSPG) with P‐meso cells resulted in a complete loss of condensation or structural organization of the cells until day14 on both heart and kidney dECM. The cells were scattered in a disorganized manner on kidney dECM and no vesicle or tubule‐like structures formed. Similarly, on heart dECM, the cells were randomly distributed, did only loosely assemble (Figure S5, Supporting Information) and no beating cells were observed. Immunostaining for markers of differentiated renal or cardiac cells did not show expression on the respective kidney and heart dECM‐HSPG on day 7 or day 14 (**Figure** **6**; Figure S6, Supporting Information). This revealed that ECM‐inductive cues essential for renal and cardiac lineage differentiation and organizational patterning are dependent on HSPG and factors bound to HS.

Our results provide a direct comparison of dECM from different tissues and conclusively show that adult mature ECM harbors tissue‐specific imprints, which support organotypic differentiation even of early fetal‐stage uncommitted precursor cells. As HSPGs themselves may alone not drive differentiation, these imprints are very likely based on heparin‐binding factors decorating the ECM in a tissue‐specific pattern. The matrix‐associated HSPGs are secreted by the tissue cells to support their own homeostasis.^1^, ^29^, ^30^, ^31^, ^32^, ^33^ We show that these HSPG‐binding factors remain to some degree in dECM preparations. When removed, no tissue‐specific differentiation was observed. The remaining ECM molecules such as collagen and laminin and the preserved physical ECM properties and architecture are alone not sufficient to induce tissue‐specific P‐meso differentiation. It is thus likely, that the degree to which the applied decellularization method preserves these HSPG‐binding factors influences the potency for specification by ECM preparations. Their comparative analysis may thus lead to the design of compositions that aid enhancement of ECM‐driven cell specification. For example, selective removal of HBGF by electrostatic intervention, which leaves HSPG intact, will allow the decoration of ECM with inductive HSPG‐binding proteins. It has been established that the patterns of ECM markings by HSPG, HBGFs, and ECM‐cleavage fragments are dynamic and change in tissues compromised by inflammation, fibrosis, and cell death.^34^ Ex vivo or in situ ECM‐modulation and cell stage selection could thus improve stem cell integration and stem cell based repair in such compromised tissues, and our model provides new means for in vitro testing of such applications.

## 3 Conclusion

Human dECM from aged, adult tissues carries factors, which directs organ‐specific differentiation of uncommitted iPSC‐derived mesoderm cells. This tissue memory is lost when HSPGs embedded in the dECM are removed. The direct comparative analysis of dECM from three different organs was possible by strict standardization and reduction of confounding variables, including the use of only human dECM, of iPSC‐derived mesoderm cells only committed to mesoderm, but not to a specific tissue fate, and of medium without inductive factors that could conceal ECM effects. The data indicate that uncommitted hiPSC‐derived cells will recognize cues in adult tissue dECM and react by tissue specification. The method and findings will allow pinpointing HSPG‐associated molecules essential for cell specification and utilization of dECM and its modification to generate functional niches for tissue engineering.

## 4. Experimental Section

##### Tissues and Cell Lines

Normal human kidney tissue was obtained after nephrectomy because of the presence of renal tumors. Normal cardiac and lung tissues were collected from explanted hearts and lung patients who underwent heart and lung transplantation. The tissues were evaluated by a pathologist and normal tissue areas were cut from the healthy region of the organs, to exclude diseased tissue. Tissues were obtained after informed consent based on approvals from the Ethics Commission of Charité with approval numbers for kidney (EA1/134/12), heart (EA4/028/12), and lung (EA2/079/13). Tissues from a total of 14 different kidneys, 5 different hearts, and 4 different lungs were obtained (Table S3, Supporting Information), cut into ≈1 cm^3^ cuboid pieces and stored at −80 °C before further processing. The human iPSC lines WISCi004‐A (IMR90), obtained from WiCell, BIHi004‐A, and BCRTi005‐A, generated at Charité, were used (information available at https://hpscreg.eu).

##### Decellularization

Tissue pieces were sectioned into 800 µm thick slices on a cryostat (Leica CM 1950) and one slice per well put into a 6‐well culture plate in distilled water. For decellularization, slices were washed with ice cold water for 2 h at 4 °C before being subjected to 0.1% w/v sodium dodecyl sulfate (SDS) (Sigma‐Aldrich), pH 7.5 at room temperature for 3 h. The detergent was changed every 30 min. Slices were washed with water at 4 °C for 30 min followed by incubation in 350 IU mL^−1^ DNase1 (Roche) in phosphate buffered saline (PBS) pH 7.5 for about 2 h at 4 °C. The slices were again washed in water at 4 °C for 30 min and incubated at 4 °C in PBS supplemented with 100 U mL^−1^ penicillin and 100 µg mL^−1^ streptomycin for 2 h. All incubation and wash steps were performed with agitation. To remove heparin sulfate proteoglycans together with the bound growth factors, dECM was treated with heparitinase‐1 solution (0.1 m sodium acetate, 10 mm calcium acetate, and 10 mU heparitinase‐1) (amsbio) for 3 h at 37 °C and washed three times each for 5 min with sterile PBS as described in ref. 14.

##### Histological Analysis

For histological and immunohistochemical analysis, tissue and dECM slices were fixed in 4% phosphate‐buffered formaldehyde solution (PFA) (Carl Roth) for 60 min at room temperature, embedded in paraffin and cut into 5 µm sections. Before staining, sections were deparaffinized and subjected to H&E staining (Sigma‐Aldrich, Carl Roth) according to established protocols.^35^ Imaging was performed using an inverse microscope (Axio Observer Z1).

##### Quantification of dECM‐Associated Heparin‐Binding Growth Factors

Native tissue, dECM, and dECM treated with heparitinase‐1 (dECM‐HSPG) were grinded into powder in liquid N2. The powder was lyophilized and the dry weight determined and dissolved in 1 mL of RIPA buffer (150 mm NaCl, 50 mm Tris, 1% TX‐100, 0.5% SDC, 0.1% SDS, pH: 7.4). Lysates were sonicated for 20 s, incubated for 24 h at 4 °C on a shaker and centrifuged at 13000 × *g* for 10 min. The following heparin‐binding growth factor concentrations were determined by enzyme‐linked immunosorbent assay (ELISA): FGF‐2, VEGF, HGF, EGF, PDGF‐BB, TGF‐ß using the respective Quantikine (R&D systems) and BMP2 (abcam) kits. All assays were performed according to the manufacturer's instructions. Absorbance was measured at 450 and 650 nm (Spectra Max 340C). Cytokine concentrations were normalized to the tissue dry weight.

##### Mesodermal and Endodermal Differentiation of Human iPSC

Human iPSC‐lines were kept in culture in TeSR‐E8 medium (Stem Cell Technologies) on geltrex (Life Technologies) coated dishes. Cells were fed daily and passaged every 4–6 days with gentle cell dissociation reagent Trypsin‐EDTA (Biochrome AG) for 5 min at 37 °C and then manually detached from the dish using a cell scraper. The resulting clumps of cells were plated in a ratio of 1:6. For hiPSC‐differentiation to mesodermal cells, the protocol established by Orlova et al. was used.^26^ Briefly, hiPSC were seeded on geltrex coated dishes and incubated in APEL‐2 (Stem Cell Technologies) and protein‐free hybridoma medium (PFHMII) (Life Technologies) and differentiation induced by the addition of CHIR99021 (1.5 µm) (Tocris), BMP4 (30 ng mL^−1^), activin A (25 ng mL^−1^), and VEGF (50 ng mL^−1^) (all from Peprotech). On day 3, the factors were removed, and cultivation continued with VEGF (50 ng mL^−1^) and the TGFβ pathway inhibitor SB431542 (10 µm) (abcam). On day 4, hiPSCs‐derived mesodermal cells (P‐meso) were harvested and used for recellularization.

To induce endoderm, hiPSC were incubated for 24 h at 37 °C in differentiation medium (RPMI1640/L‐Glu, B27, 100 ng mL^−1^ activin A, 3 µm CHIR99021, 10 µm ROCK‐Inhibitor), washed twice in 2 KnockOut‐DMEM‐F12, passaged using Accutase. Single cells (2.0 × 10^5^ cells cm^−2^) were subsequently incubated in differentiation medium for 24 h at 37 °C/5% CO_2_ Incubator. Medium was subsequently supplemented with 0.5 mm sodium butyrate, and cells further incubated for 2–6 days at 37 °C/5% CO_2_ with daily medium change.

##### Recellularization of dECM with P‐meso Cells

Approximately 800 µm thick slices of kidney, heart, and lung dECM and heparitinase‐1 treated dECM, were placed on hydrophobic floating membranes (Whatman) in a six‐well plate to provide an ALI condition (Figure S2f, Supporting Information). To analyze single matrix proteins and geltrax, membranes were coated with the individual matrix proteins Collagen IV (Sigma‐Aldrich), laminin (Biolamina), fibronectin (Corning), and geltrex (Life Technologies). 500 000 hiPSC‐meso cells were placed on the different matrix preparations in APEL‐2 + PFHMII. Medium was supplemented with Rock Inhibitor Y‐27632 (WAKO) for the initial 24 h. Cultivation continued for up to 14 days in APEL‐2 + 5% PFHMII and medium was changed every 48 h. For histology and immunohistochemistry, cells were fixed at days 3, 7, and 14 in 4% PFA at room temperature for 1 h and embedded in paraffin for further analysis.

##### Immunohistochemistry

For immunostaining, 5 µm paraffin sections cut from fixed dECM or recellularized dECM were deparaffinized and subjected to antigen retrieval solution (DAKO). The sections were permeabilized with 0.1% TX‐100 in PBS pH 7.4 (T‐PBS) three times for 5 min, blocked for 10 min with 1% bovine serum albumin (BSA) (Sigma‐Aldrich) in T‐PBS and for 60 min with 5% donkey serum (Merck Millipore), and 1% BSA in PBS before immunostaining. Primary antibodies were applied overnight at 4 °C; all antibodies were diluted in 5% donkey serum and 1% BSA in PBS. The sections were then washed with T‐PBS three times for 5 min each, and incubated with secondary antibodies for 1 h at room temperature. Finally, after washing in TPBS three times for 5 min each, sections were mounted with immunoselect‐antifading mounting medium including 4′,6‐diamidino‐2‐phenylindole (DAPI) (Dianova). The same protocol was used for negative control staining, except that the primary antibody was omitted. For a list of the antibodies used, see Table S1, Supporting Information. To detect VEGF, anti VEGF (Santa Cruz Biotechnology Inc.) and secondary antibody IgG H&L (horse‐radish peroxidase, HRP) preadsorbed (abcam) were used, followed by 3,3′‐diaminobenzidine (DAB)/plus (abcam) chromogen detection. To visualize structures, the sections were counterstained with Masson trichrome (Sigma‐Aldrich) using a standard protocol.^35^ Imaging was performed using either an inverse microscope (Axio Observer Z1, Carl Zeiss) or the Operetta high content imager and Columbus image analysis server (both PerkinElmer).

##### Quantitative Real‐Time Polymerase Chain Reaction (qPCR)

RNA was extracted from recellularized dECM cultures at days 7 and 14 using picopure RNA isolation kit (ThermoFisher Scientific) and cDNA was prepared by TaqMan Reverse Transcription Reagents (ThermoFisher Scientific). The components of SensiFAST SYBR Hi‐ROX (Bioline), were mixed with the yielded cDNA. Specific genes were amplified by the application of primers listed in Table S2, Supporting Information. For each primer an additional negative control was applied (without cDNA). The real‐time PCR‐QuantStudio 6Flex (Applied Biosystems, Life Technologies) was used and run with 40 cycles.

##### Functional Assay of dECM‐Induced Proximal Tubular Epithelial Cells

Electrolyte reabsorption assays were performed using NaCl as electrolytes to examine functional properties of cells expressing renal proximal tubule epithelial cell markers. Cellular sodium green (ThermoFisher Scientific) uptake was evaluated as described.^36^ Recellularized kidney dECM at day 14 of cultivation was incubated with 10 mm sodium green in 90 mm NaCl, 60 mm *N*‐methyl‐*d*‐glucamine, 2 mm NaH_2_PO_4_, 5 mm KCl, 1 mm CaCl_2_, 1.2 mm MgSO_4_, 32 mm 4‐(2‐hydroxyethyl)‐1‐piperazineethanesulfonic acid (HEPES), 10 mm glucose at pH 7.4 for 60 min at room temperature, and washed with PBS. To assess specificity of cellular sodium uptake, cells were incubated with 50 µm ouabain (Sigma‐Aldrich) for 1 h to restrain Na/K ATPase. The incubated samples were washed with PBS and fixed in 4% PFA, nuclei were stained with DAPI and uptake of sodium was visualized by fluorescence microscopy (Axio Observer Z1, Carl Zeiss).

##### Statistical Analysis

Quantitative results are reported as mean ± standard error of the mean (SEM). Statistical comparisons were performed using unpaired *t*‐tests, unless specified otherwise. For multiple comparisons of more than two groups, one‐way ANOVA was used with Tukey's multiple comparison post hoc tests for significance. GraphPad Prism 5 (GraphPad Software, La Jolla, USA) was used for statistical analysis. *p* < 0.05 was considered statistically significant.

## Conflict of Interest

The authors declare no conflict of interest.

## Author Contributions

I.U. and A.K. conceptualized the study; I.U., J.F.B., A.R., B.E., C.S., C.K., S.H., P.R. and A.K. provided the methodology; I.U. and A.K. performed the analysis and investigation; I.U., J.F.B., A.R., B.E., C.S., C.K., S.H. and A.K. provided resources and materials; I.U. and A.K. drafted the manuscript; I.U., J.F.B., A.R., C.K., S.H., P.R., A.K. reviewed and edited the draft, with input from all authors; J.F.B., P.R. and A.K. provided supervision to the experiments; A.K. managed funding acquisition.

## Supporting information

Supporting Information

Supplemental Video 1
