# Supplementary material for: Adult Tissue Extracellular Matrix Determines Tissue Specification of Human iPSC‐Derived Embryonic Stage Mesodermal Precursor Cells
Source: Adv Sci (Weinh). 2020 Jan 21;7(5):1901198. doi: 10.1002/advs.201901198 (PMC7055561; doi:10.1002/advs.201901198)
Supplement: Supplementary file 1 — Supporting Information [file ADVS-7-1901198-s001.pdf]

Copyright WILEY-VCH Verlag GmbH & Co. KGaA, 69469 Weinheim, Germany, 2020.

ADVANCED SCIENCE
Open Access

Supporting Information

for *Adv. Sci.,* DOI: 10.1002/advs.201901198

Adult Tissue Extracellular Matrix Determines Tissue Specification of Human iPSC-Derived Embryonic Stage Mesodermal Precursor Cells

*Imran Ullah, Jonas Felix Busch, Anja Rabien, Bettina Ergün, Christof Stamm, Christoph Knosalla, Stefan Hippenstiel, Petra Reinke, and Andreas Kurtz**

SUPPLEMENTARY FIGURES AND TABLES

Supplementary Video S1 (see in the supporting information)

Beating cardiomyocytes develop from P-meso cells at day 7 post seeding only on heart dECM.

Figure S1

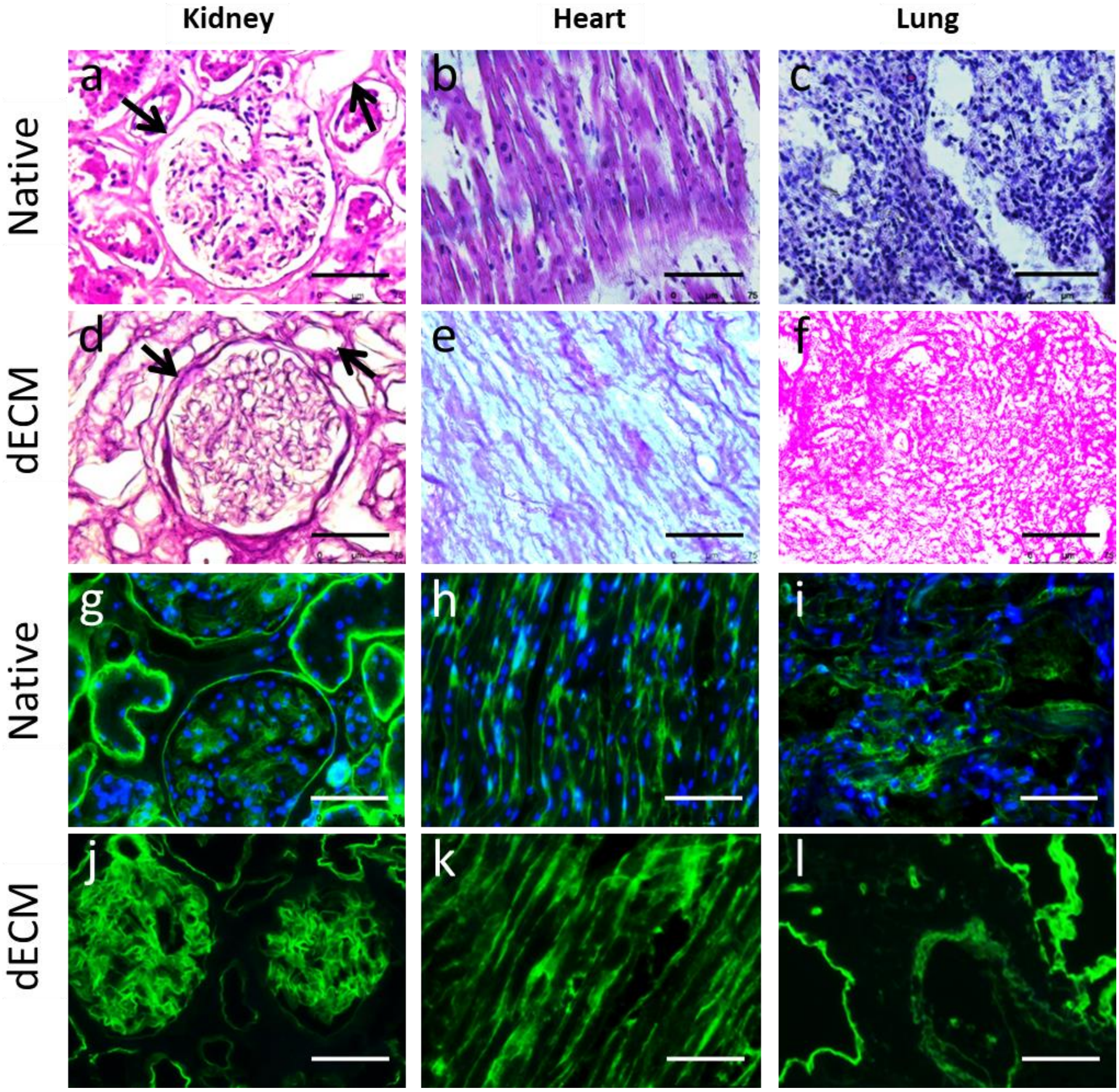

Figure S1. Characterization of decellularized human kidney, heart and lung tissue slices. (**a-f**) H&E staining of a section of human renal, cardiac and lung tissue of native (upper panel; **a,b,c**) and decellularized tissue (lower panel; **d,e,f**). (**g-l**) Laminin (green) and nuclei (blue) staining in native kidney, heart and lung tissues (**g,h,i**) and corresponding decellularized tissues (**j,k,l**). Scale bar: 75µm

Figure S2

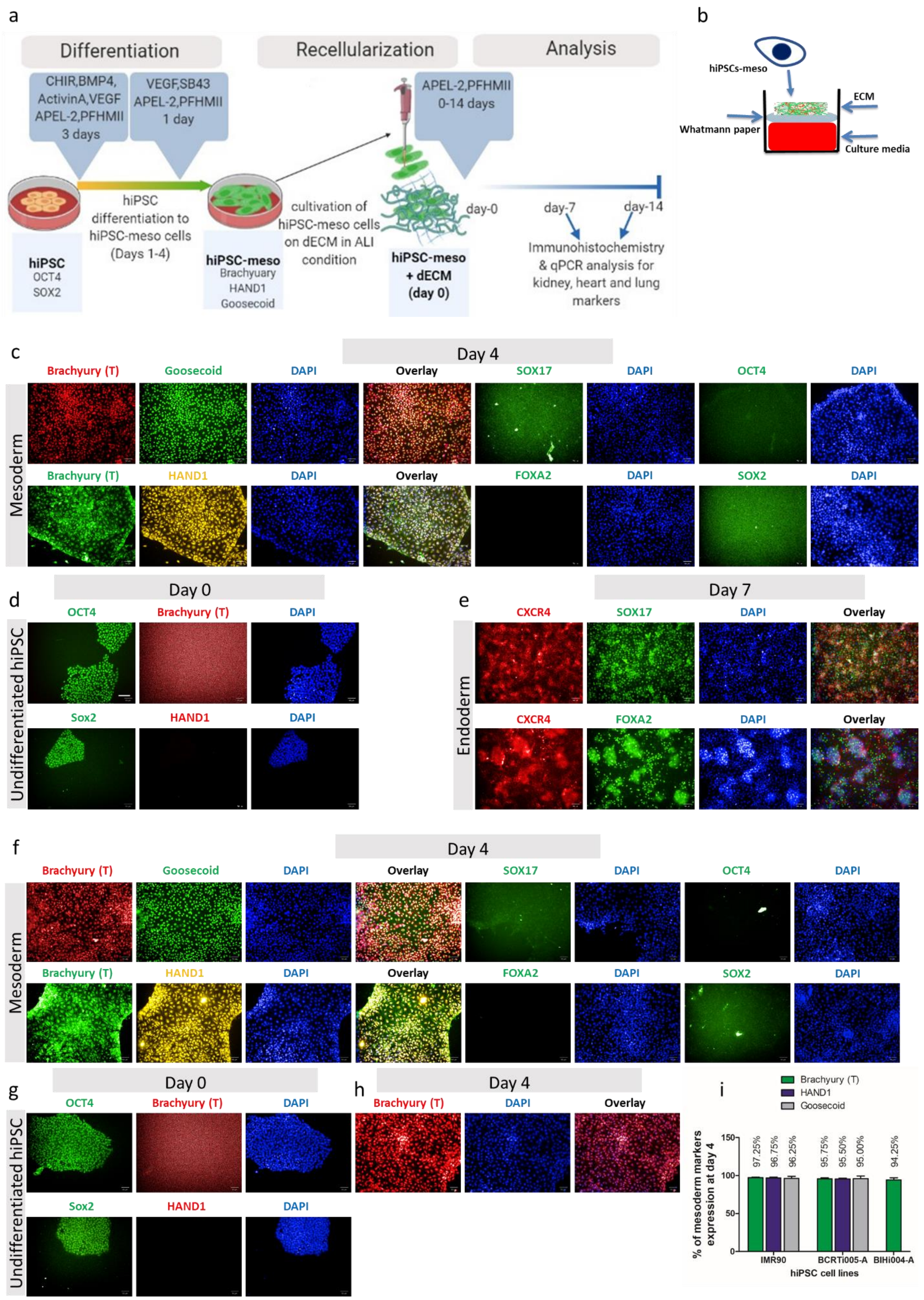
a
Differentiation
Recellularization
Analysis
CHIR,BMP4, ActivinA,VEGF APEL-2,PFHMII 3 days
VEGF,SB43 APEL-2,PFHMII 1 day
APEL-2,PFHMII 0-14 days
hiPSC differentiation to hiPSC-meso cells (Days 1-4)
cultivation of hiPSC-meso cells on dECM in ALI condition
day-0
day-7
day-14
hiPSC
OCT4
SOX2
hiPSC-meso
Brachyuary
HAND1
Goosecoid
hiPSC-meso + dECM (day 0)
Immunohistochemistry & qPCR analysis for kidney, heart and lung markers
b
hiPSCs-meso
ECM
Whatmann paper
Culture media
c
Day 4
Mesoderm
Brachyury (T)
Goosecoid
DAPI
Overlay
SOX17
OCT4
HAND1
FOXA2
SOX2
d
Day 0
Undifferentiated hiPSC
OCT4
Brachyury (T)
DAPI
Sox2
HAND1
e
Day 7
Endoderm
CXCR4
SOX17
DAPI
Overlay
FOXA2
f
Day 4
Mesoderm
Brachyury (T)
Goosecoid
DAPI
Overlay
SOX17
OCT4
HAND1
FOXA2
SOX2
g
Day 0
Undifferentiated hiPSC
OCT4
Brachyury (T)
DAPI
Sox2
HAND1
h
Day 4
Brachyury (T)
DAPI
Overlay
i
Brachyury (T)
HAND1
Goosecoid
% of mesoderm markers expression at day 4
150
100
50
0
97.25%
96.75%
96.25%
95.75%
95.50%
95.00%
94.25%
IMR90
BCRTi005-A
BIHi004-A
hiPSC cell lines

Figure S2. Differentiation, re-cellularization scheme and characterization of human iPSC derived mesodermal cells (P-meso). (**a**) Schematic presentation of time line and media composition used for mesodermal induction of hiPSC (Differentiation section) and seeding conditions and time points of analysis of P-meso cells on dECM preparations (Recellularization and analysis section). **(b)** Schematic depiction of air-liquid interphase cultivation model. The assays were performed with three hiPSC – lines (WISCi004-A, BCRTi005-A, BIHi004-A). **(c-e)** Data obtained with hiPSC-line WISCi004-A (IMR90): (**c**) Characterization of hiPSC-derived mesodermal cells (P-meso) at day 4 post differentiation induction. Cells express mesodermal markers Brachyury (T), HAND1 and goosecoid, but not endodermal markers FOXA2 and Sox17 nor pluripotency markers Oct4 and Sox2. **(d,e)** To show utility of the used detection methods, expression of pluripotency and mesodermal markers was analysed in undifferentiated hiPSC, and of endodermal markers in hiPSC-derived endodermal cells. **(d)** Oct4 and Sox2 are expressed in undifferentiated hiPSC. **(e)** Endodermal markers Sox17, FOXA2 and CXCR4 are expressed in hiPSC – derived endodermal cells at day 7 post endodermal induction. **(f,g)** Data from hiPSC - line BCRTi005-A: **(f)** hiPSC-derived mesodermal cells (P-meso) at day 4 post differentiation express mesodermal markers Brachyury (T), HAND1 and goosecoid, but not endodermal markers FOXA2 and Sox17 nor pluripotency markers Oct4 and Sox2. **(g)** Oct4 and Sox2 are expressed in undifferentiated hiPSC. **(h)** Data confirmed with hiPSC-line BIHi004-A with hiPSC-derived mesodermal cells (P-meso) at day 4 post differentiation express mesodermal markers Brachyury (T). **(i)** Quantitative analysis of mesoderm induction based on T, HAND1 and Goosecoid expression at day 4 post differentiation. Scale bar: 50µm. Mean ± SEM, n=6 (two for each cell line).

Figure S3

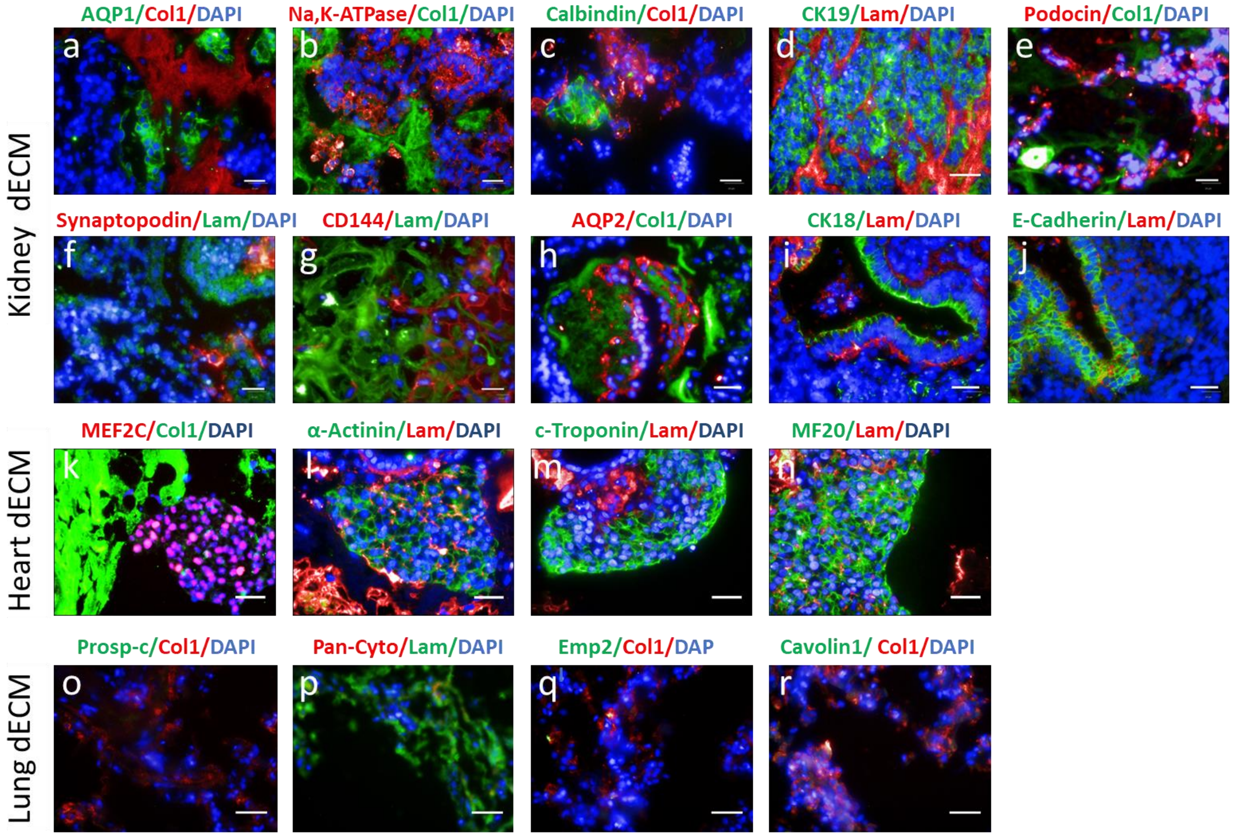

Figure S3. Characterization of P-meso cells at day 7 post seeding on kidney, heart and lung dECM. Kidney and heart cell markers were selected to identify different renal cell types (**a-j**) and immature and mature cardiac cells (**k-n**): (**a,b**) AQP1 and Na,K-ATPase for proximal tubules, (**c**) Calbindin for distal tubules (**d**) CK19 for loop of Henle, (**e,f**) podocin and synaptopodin for glomerulus, (**g**) CD144 for endothelial cells, (**h**) AQP2 for collecting duct, (**i,j**) CK18 and E-Cadherin for nephron epithelia. (**k-n**) MEF2C as cardiac progenitor marker, **(l-n)** α-actinin, c-Troponin and MF20 as more mature cardiomyocyte markers. (**o-r**) none of the tested lung cell markers are expressed on lung dECM. Images are from different depths of the 800µm thick ECM showing uniform cellular penetration. Scale bars: 20µm

Figure S4

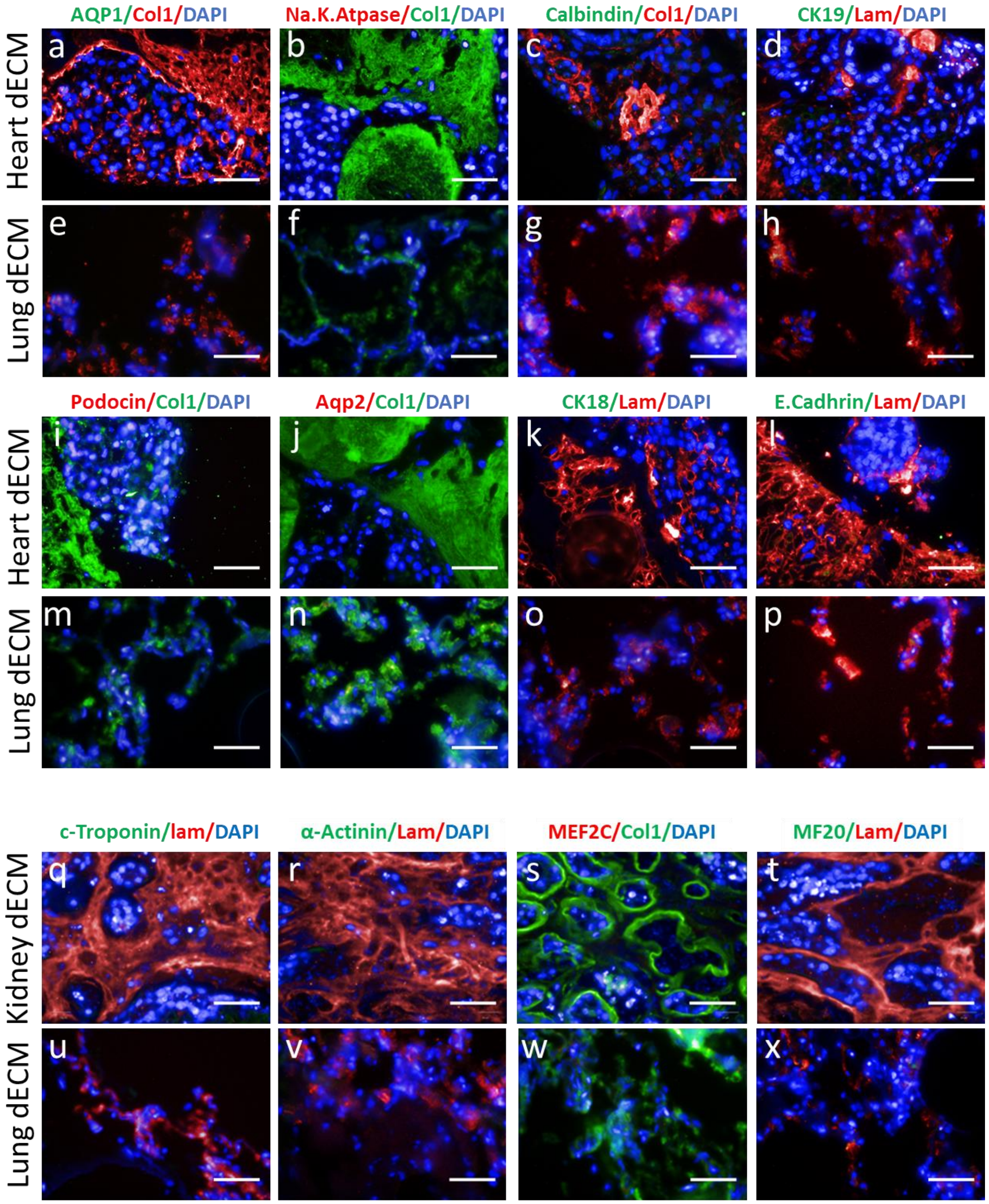

Figure S4. Expression of tissue specific markers in P-meso cells is restricted to the source tissue of dECM and not detectable on ectopic dECM. Human iPSC-meso cells were seeded on kidney, heart and lung ECM and expression of markers for kidney, heart and lung cell types

was assessed on the ectopic matrices. Expression of kidney markers by P-meso cells was not detectable on days 7 (not shown) or day 14 when seeded on heart dECM (**a-h**), or on lung dECM (**i-p**). Similarly, no expression of cardiac markers was detected in P-meso cells seeded on kidney dECM on day (**q-t**) or on lung dECM (**u-x**). Images are from different depths of the 800μm thick ECM showing uniform cellular penetration. Scale bar: 20μm

Figure S5

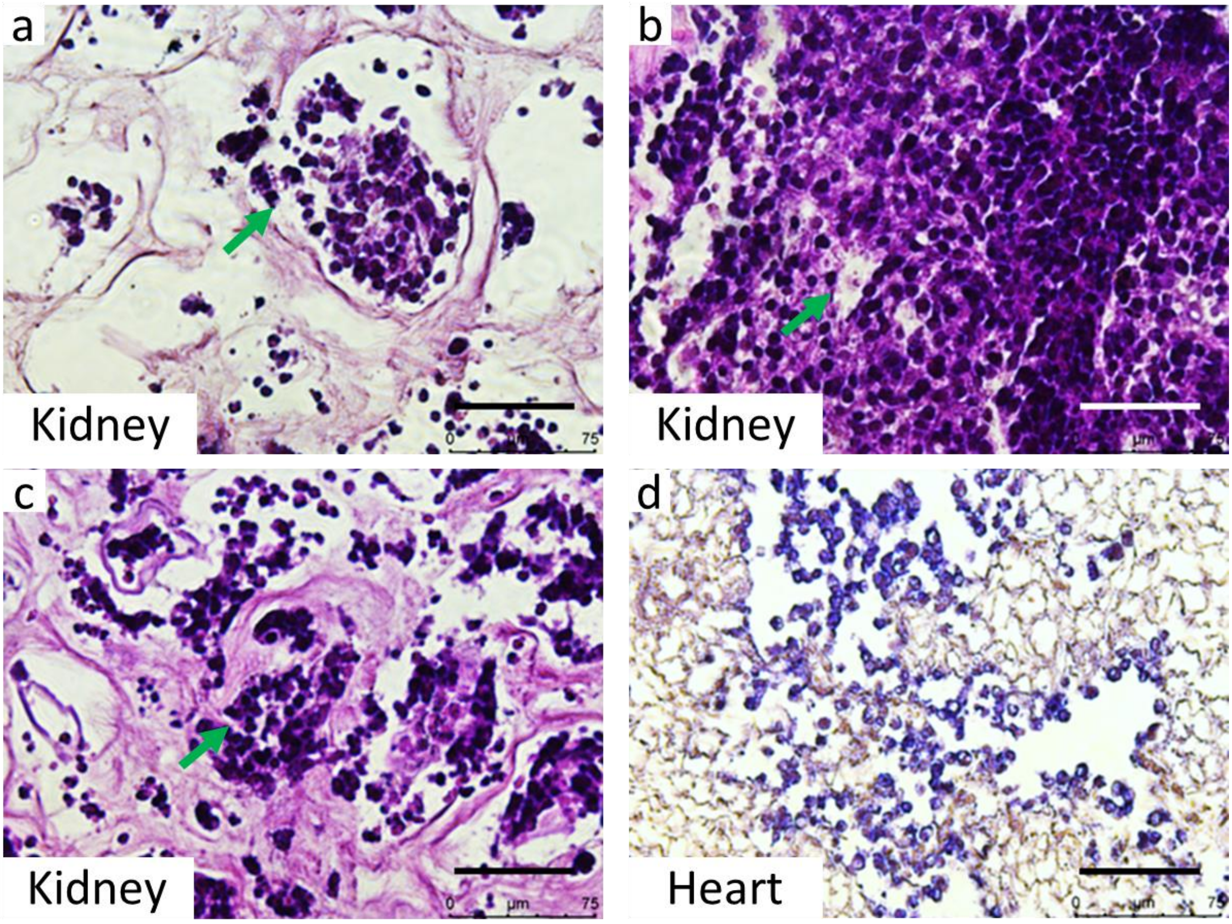

Figure S5. Histological characterization of kidney, heart and lung dECM-HBGF repopulated with P-meso cells. Representative hematoxylin and eosin (H&E) staining of kidney and heart dECM-HSPG sections at day 3, day 7 and day 14 post seeding with P-meso cells. (**a-c**) P-meso cells seeded on kidney and (**d**) on cardiac dECM-HSPG. (**a**) Cells form a cluster like aggregate on a glomerular structure (arrow). (**b**) Cells are generally disorganized and scattered on scaffold until day 14 (**c**) Cells close to the tubules/vessel - like structures (arrow) do not arrange as observed in non-heparitinase treated dECM (see figure 1). (**d**) On heart dECM-HSPG, the cells are distributed evenly, and the cells do not assemble into tightly packed clusters as observed in non-heparitinase treated dECM (see figure 1). Scale bar: 75µm

Figure S6

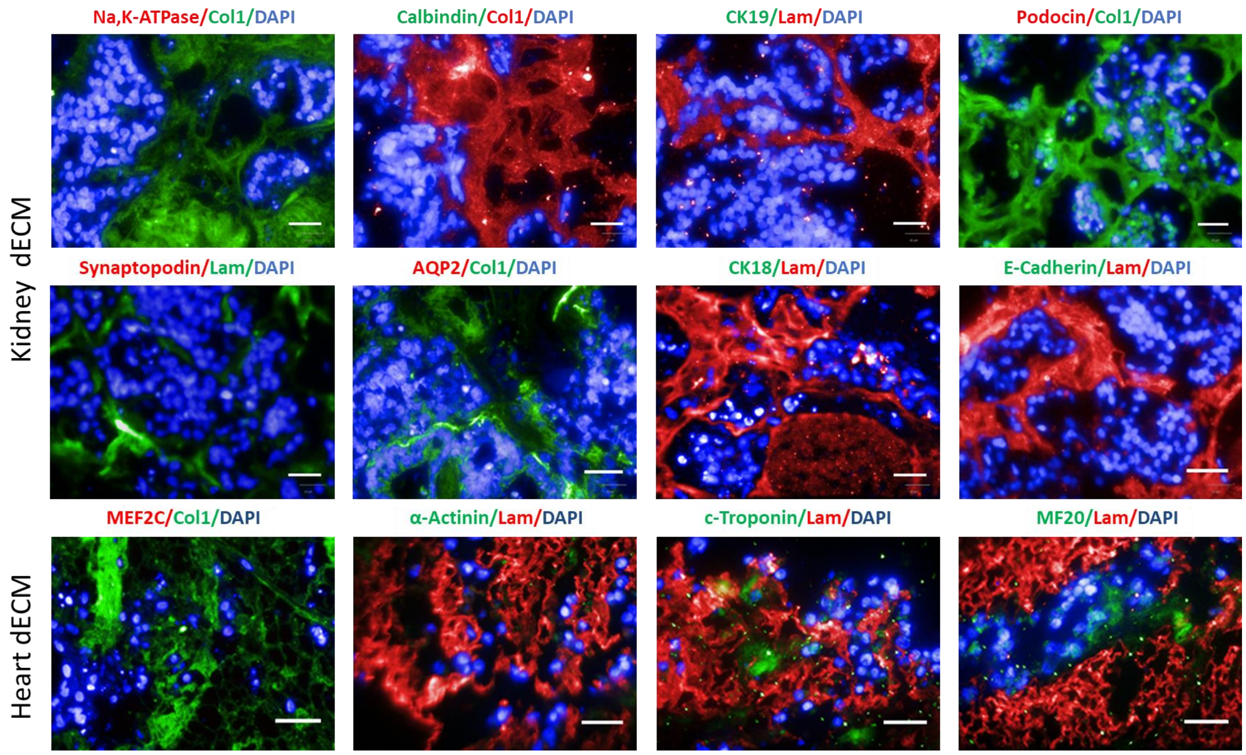

Figure S6. Tissue specific differentiation of P-meso cells was not induced by dECM-HSPG. None of the indicated renal or cardiac specific cell markers were detected by immunohistochemistry in P-meso cells seeded on kidney or heart dECM-HSPG.

Supplementary Table 1: List of antibodies used for Immunofluorescence

**Name**	**Species**	**Description**	**Company**	**Cat.Nr**
AQP 1	Rabbitpolyclonal	Proximal tubule	Proteintech	20333-1-AP
Na,K-ATPase	Rabbitpolyclonal	Proximal tubule	Abcam	AB58475
CK19	Mouse monoclonal	Loop of Henle	Santa Cruz	SC-6278
Calbindin	Rabbitpolyclonal	Distal tubule	Temecula California	AB1778
NPHS2	Rabbitpolyclonal	Podocyte	Abcam	ab50339
SYNPO	Mouse monoclonal	Podocyte	Progen	65294
CD144	Mouse monoclonal	Endothelial cells	BD Biosciences	555661
Aqp2	Rabbit polyclonal	Collecting duct	Novus Biologicals	NB110-74682
CK18	Mouse monoclonal	Whole nephron	Santa Cruz	SC -6259
E-Cadherin	Mouse monoclonal	Whole nephron	BD Biosciences	610181
Alpha actinin	Mouse monoclonal	Cardiac actinin	Sigma	A7811
MF-20	Mouse monoclonal	Myosin filament	DSHB	AB_2147781
RV-C2	Mouse monoclonal	Cardiac troponin	DSHB	NS0
MEF2C	Rabbitpolyclonal	Myocyte enhancer	CellSignaling technology	5030
Anti proSP-C	Rabbitpolyclonal	Type II Pneumocyte	Millipore	AB3786
Anti Caveolin1	Rabbitpolyclonal	Type I Pneumocyte	Santa Cruz	SC-894
EMP2	Rabbitpolyclonal	Type I Pneumocyte	Sigma	HPA014711
pan-Cytokeratin	Mouse monoclonal	Epithelial	Santa Cruz	sc-8018
EG-VEGF	Goatpolyclonal	VEGF marker	Santa Cruz	SC-30343
Laminin	Rabbitpolyclonal	ECM laminin	Abcam	Ab11575
Col1	Mouse monoclonal	ECM Collagen	Abcam	ab6308
OCT4	Mouse conjugated	Pluripotency	BD Bioscience	560217
SOX2	Mouse conjugated	Pluripotency	BD Bioscience	560292
Brachyuary	Rabbit Polyclonal	Definitive mesoderm	Santa Cruz	SC-20109
Brachyuary	Mouse monoclonal	Definitive mesoderm	Santa Cruz	SC-166962
HAND1	Rabbit polyclonal	Definitive mesoderm	Abcam	Ab196622
Goosecoid	Goat polyclonal	Definitive mesoderm	R&D systems	AF4086

CXCR4-APC	Mouse conjugated	Definitive endoderm	Miltenyi Biotec	130-098-357
FOXA2	Goat conjugated	Definitive endoderm	R&D systems	IC2400G
SOX17	Mouse monoclonal	Definitive endoderm	R&D systems	MAB1924

Supplementary Table 2: List of Primer used for qPCR

Target	Description	FORWARD PRIMER	REVERSE PRIMER
AQP1	Proximal tubule	AAGCTCTTCTGGAGGGCAG	CACCTTCACGTTGTCCTGGACCG
NCCT	Distal tubule	CACCAAGAGGTTTGAGGACAT	GACAGTGGCCTCATCCTTGA
Na,K-Atpase	Proximal tubule	GCTGACCCGCCATCGCCA	ACCAACTGCCACCGGTCCT
NKCC2/SLC12A1	Loop of Henle	TTTGGAGCTGTTTTGTGCTG	ATGGGTCCCCCTGTTAAGAC
CK-7	Collecting duct	TCCGCGAGGTCACCATTAAC	GCTCTGTCAACTCCGTCTCAT
Podocin	Podocyte	CCTGCCTGGATACCTACCACA	GCACAGCTTTAGATACATGAGCA
E-Cadherin	Whole nephron	GAGGACCAGGACTTTGACTT	AGATACCGGGGGACACTCA
CD144	Endothelial cells	AACTTCCCCTTCTTCACCC	AAAGGCTGCTGGAAAATG
MEF2C	Cardiac progenitor	CGAAACTCACCAGGTCTGCT	TAGCCAATGACTGAGCCGAC
GATA4	Cardiac progenitor	CAACTGCCAGACCACCACC	CCCTCTTTCCGCATTGCAAG
Nkx2.5	Cardiac progenitor	CCCGCCTTCTATCCACGTG	GCCTCTGTCTTCTCCAGCTC
b-MHC	Cardiac mature	GAAGCCCAGCACATCAAAAG	GATCACCAACAACCCCTACG
cTNI	Cardiac mature	TTTGACCTTCGAGGCAAGTTT	CCCGGTTTTCCTTCTCGGTG
MLC-2V	Cardiac mature	TGAGAGACACCTTTGCTGCC	CTCCCCAAACATTGTGAGGAAC
GAPDH	Control	TTGCCATCAATGACCCCTTCA	CGCCCCACTTGATTTTGGA
HPRT	Control	AGTCTGGCTTATATCCAACACTTCG	GACTTTGCTTTCCTTGGTCAGG

Supplementary Table 3: Age and sex of tissue donors

Kidney		Heart		Lung	
**Age**	**Sex**	**Age**	**Sex**	**Age**	**Sex**
74	m	64	m	39	m
72	m	64	m	42	m
56	f	58	m	71	f
81	m	52	m	69	m
61	m	70	f		
55	m				
68	m				
63	m				
65	m				
54	f				
64	m				
66	f				
44	m				
84	m				
